# The Arabidopsis Mutant of the Small Intrinsically Disordered Protein DSS1(V) Exhibits Increased Sensitivity to Drought Stress

**DOI:** 10.1002/pld3.70140

**Published:** 2026-02-05

**Authors:** Ivana Nikolic, Maja Sabljic, Mira Milisavljevic, Ivan Radin, Gordana Timotijevic

**Affiliations:** ^1^ Institute of Molecular Genetics and Genetic Engineering, University of Belgrade Group for Plant Molecular Biology Belgrade Serbia; ^2^ Project Management Analyst Belgrade Serbia; ^3^ Department of Plant and Microbial Biology University of Minnesota St. Paul Minnesota USA

**Keywords:** *Arabidopsis thaliana*, drought, intrinsically disordered proteins, protein homeostasis

## Abstract

Drought has emerged as one of the most severe and widespread environmental stresses affecting plants. Crops exposed to varying levels of drought, ranging from moderate to severe, often experience notable declines in yield or reduced harvest quality. Investigating the molecular mechanisms and cellular factors involved in plant defense against drought is crucial—not only for advancing our understanding of these processes but also for ensuring sustainable food production and supporting humanity's survival. Our previous work identified the small intrinsically disordered protein DSS1 (deleted in split‐hand/split‐foot) as a key factor in the stress defense mechanisms of *Arabidopsis thaliana* . The absence of DSS1(V) led to increased sensitivity of plants to oxidative stress induced by hydrogen peroxide or methyl viologen. As drought can induce oxidative stress in plant cells, we investigated if DSS1(V) protein can mitigate stress caused by mild to moderate drought. Alongside the wild‐type (WT) strain, the analysis included knockout plants lacking the DSS1(V) gene and plants overexpressing this gene. Various stress‐related parameters, including lipid peroxidation, total phenol content, chlorophyll levels, and protein oxidation, were measured. Results indicated that the DSS1(V) knockout line displayed significantly higher sensitivity to drought compared to WT plants. However, elevated levels of DSS1(V) transcripts in the overexpressing lines did not confer a protective effect, as these lines did not exhibit reduced drought sensitivity. These findings provide compelling evidence highlighting the critical involvement of the DSS1(V) protein in the mechanisms underlying plant responses to environmental stress, particularly water deficiency. This protein appears to enable plants to cope with the challenges posed by drought conditions, emphasizing its importance in maintaining cellular homeostasis and mitigating the adverse effects of water scarcity.

## 1 Introduction

Abiotic stress refers to adverse conditions caused by non‐living environmental factors, which negatively impact plant growth and development. Salinity, drought, flooding, extreme temperatures, nutrient deficiencies, and toxic heavy metals can significantly reduce the yield of economically important plant species, posing a substantial threat to agriculture. Furthermore, abiotic stress presents a major challenge to global food security, highlighting the critical need to understand plant response mechanisms to develop effective mitigation strategies.

A hallmark of all abiotic stresses is the excessive accumulation of reactive oxygen species (ROS) within plant cells (You and Chan 2015). ROS induce cellular damage by disrupting biomolecules and impairing essential physiological processes (Das and Roychoudhury 2014), ultimately compromising plant health and productivity. This process occurs across various organelles, including chloroplasts, mitochondria, plasma membrane, cell wall, and peroxisomes (Apel and Hirt 2004; Phua et al. 2021). ROS comprise numerous oxygen‐derived molecules, such as peroxides and free radicals, which exhibit high reactivity due to the presence of an unpaired electron in their valence shell (Gaur et al. 2020). At low concentrations, they act as signaling molecules, including in stress responses (Vranová et al. 2002), but excessive accumulation causes cellular damage, affecting membranes, nucleic acids, lipids, and proteins (Teotia and Singh 2014). To maintain ROS homeostasis and safeguard cellular integrity, plants utilize diverse antioxidant defense systems, consisting of both enzymes and non‐enzymatic metabolites (Dumanović et al. 2021). Plants, as sessile organisms, have evolved adaptive mechanisms to withstand drought conditions (Cruz de Carvalho 2008). A key consequence of water scarcity is increased ROS production, leading to oxidative stress (Cruz de Carvalho 2008). Prolonged drought eventually results in oxidative damage to plant cells, tissues, and organs (Smirnoff 1993). Under stress, electrons are transferred from ferredoxin to O₂ instead of NADP (Meller's reaction), accelerating ROS production. This effect intensifies during drought as stomatal closure lowers CO₂ levels, preventing NADPH oxidation (Barickman et al. 2021). During the early stages of drought, the stable ROS levels become disrupted, increasing as stomatal closure triggers signal transduction and associated physiological responses (Cruz de Carvalho 2008). However, prolonged drought exacerbates ROS accumulation beyond the capacity of antioxidant defenses, leading to oxidative damage and, ultimately, cell death (Cruz de Carvalho 2008).

Climate change is a major driver of drought worldwide (Rana 2013), though other factors such as high temperatures, intense light, and dry winds also contribute (Dai 2013; Lisar et al. 2012). However, drought is not always due to water scarcity; in some cases, plants struggle to absorb available water due to cold, salinity, or flooding, a condition known as physiological drought. Its impact depends on species, developmental stage, and environmental conditions (Salehi‐Lisar and Bakhshayeshan‐Agdam 2016). Drought affects the growth and development of plants in different ways, often negatively affecting their yield, especially in crops (Salehi‐Lisar and Bakhshayeshan‐Agdam 2016). Drought symptoms include wilting, leaf drop, stunted growth, branch cracking, and, in severe cases, plant death (Farooq et al. 2009). It disrupts cell division, elongation, and differentiation due to turgor loss, impaired enzyme activity, and reduced photosynthetic efficiency (Shao et al. 2008). Leaf anatomy adapts with smaller leaves, thicker cell walls, fewer stomata, and cuticle formation (Salehi‐Lisar and Bakhshayeshan‐Agdam 2016; Chernyad'ev 2005). Drought also impairs nutrient uptake, often causing nitrogen and phosphorus deficits while leaving potassium levels unaffected (Motahari et al. 2014; Akhtar and Naveela Nazir 2013). Abscisic acid (ABA) plays a key role in drought response by triggering stomatal closure, reducing water loss, and limiting CO₂ absorption, ultimately lowering photosynthesis (Salehi‐Lisar and Bakhshayeshan‐Agdam 2016).

Plants employ diverse mechanisms to defend against drought‐induced stress, and accumulating evidence indicates that numerous intrinsically disordered proteins (IDPs) play pivotal roles in modulating these responses. For instance, the stress and growth interconnector (SGI) interacts with other drought‐responsive proteins such as dehydrins and catalase isoforms, stabilizing them and enhancing their ROS‐scavenging activity (Liu et al. 2023). Similarly, the salt tolerance related protein (STRP), particularly characterized in *A. thaliana* , is an IDP involved in responses to abiotic stresses including cold, salinity, and drought. Its structural flexibility enables interactions with multiple signaling partners, and it has been implicated in ABA signaling, transcriptional regulation, and protein stabilization under stress conditions (Fiorillo et al. 2023). Moreover, various late embryogenesis abundant (LEA) proteins, many of which are IDPs, accumulate in cells during drought stress, functioning as molecular shields that stabilize membranes, protect enzymes, and safeguard other proteins against dehydration and associated damage (Magwanga et al. 2018).

In addition, highly conserved DSS1 (Deletion of SUV3 Suppressor 1) proteins play important roles in plant stress response, namely, they have a crucial function in maintaining protein homeostasis and facilitating protein degradation under stress conditions (Zhang et al. 2014). DSS1s are intrinsically disordered proteins (IDP), lacking a defined 3D structure (Paraskevopoulos et al. 2014; Babu et al. 2011). They are multifunctional and can interact with various proteins in different complexes and contribute to numerous biological processes (Kragelund et al. 2016). Even when bound to a specific complex, certain regions of DSS1 remain disordered, resulting in limited secondary structure, which depends on the target complex (Ellisdon et al. 2012; Kragelund et al. 2016). Their sequences contain negatively charged residues and three conserved hydrophobic regions flanked by acidic residues (Kragelund et al. 2016).

DSS1 proteins interact with the 26S proteasome, aiding its assembly and ubiquitin recognition (Krogan et al. 2004; Paraskevopoulos et al. 2014). The 26S proteasome comprises a 20S proteolytic core and two 19S regulatory complexes that recognize and transport ubiquitinated proteins (Kragelund et al. 2016). In yeast ( *Saccharomyces cerevisiae* ), DSS1 stabilizes the 19S complex by recruiting subunits Rpn3 and Rpn7 (Tomko and Hochstrasser 2014). Beyond the proteasome, yeast DSS1 interacts with Thp1 in the TREX‐2 complex for nuclear mRNA export (Ellisdon et al. 2012) and with Csn12‐Thp3 in mRNA splicing and export (Wilmes et al. 2008). In humans, DSS1 associates with BRCA2 to mediate homologous recombination in DNA repair (Yang 2002), binds RPA (Zhao et al. 2015), and integrates with the RNA polymerase II complex (Baillat et al. 2005). Yeast studies further suggest a role in oxidative stress, where DSS1 marks oxidized proteins for removal via DSSylation (Zhang et al. 2014). Proteins are highly vulnerable to free radical damage (Sitte 2003), and DSS1‐protein fusions require ATP but are inhibited by EDTA or heat, indicating an ATPase‐dependent process (Zhang et al. 2014). Unlike ubiquitin, DSS1 binds targets through four residues—W27, W39, W43, and F52 (Zhang et al. 2014)—and may facilitate their ubiquitination and degradation via specific E3 ligases (Zhang et al. 2014). DSS1 is highly conserved across eukaryotes, suggesting an ancient, universal defense mechanism (Zhang et al. 2014). The involvement of DSS1 proteins in the assembly and functioning of such a wide range of complexes highlights their pronounced multifunctionality.

In the genome of *A. thaliana* , two DSS1 proteins have been identified: a longer DSS1(I) (accession number At1g64750) and a shorter DSS1(V) (At5g45010), located on chromosomes I and V, respectively (Dray et al. 2006). Analysis of knockout DSS1 mutants, *dss1(I)* and *dss1(V)*, indicated functional differences between these genes, as their single mutants exhibited distinct phenotypes and developmental dynamics compared to WT plants (I. P. Nikolić et al. 2021; I. Nikolić et al. 2023). The two DSS1 proteins differentially influenced protein homeostasis, as demonstrated by varying mutant sensitivities to oxidative stress (I. Nikolić et al. 2023). DSS1(V) deficiency led to the accumulation and inefficient removal of oxidized proteins, whereas DSS1(I) disruption had a limited impact on this process. Additionally, a functional complementation test using *a Ustilago maydis dss1* mutant line sensitive to genotoxic agents showed that DSS1(I) from *A. thaliana* can restore the WT phenotype of *U. maydis* , while DSS1(V) does not, suggesting that DSS1(I) plays an essential role in homologous recombination (I. Nikolić et al. 2024).

Building on our previous findings that DSS1(V) is involved in the response to oxidative stress agents such as hydrogen peroxide and methyl viologen, this study investigates its potential role in Arabidopsis defense mechanisms under drought conditions—a more ecologically relevant environmental stressor. By assessing various physiological parameters, we examined the drought sensitivity of the DSS1(V) knockout mutant and the possible resistance of the DSS1(V) overexpression plant line. The results clearly highlight DSS1(V) as a key player in molecular stress defense, as the knockout line exhibited increased sensitivity to drought. These findings underscore DSS1(V)’s significance in plant stress physiology and its potential implications for improving crop resilience to abiotic stress factors.

## 2 Material and Methods

### 2.1 Plant Material and Growing Conditions

The seeds of *A. thaliana* , ecotype Columbia‐0 (Col‐0), were used in this experiment. In addition to wild‐type (WT) plants, two mutants were used: a knockout *dss1(V)* line (KO‐*dss1(V)*, previously generated (I. Nikolić et al. 2023) and a line overexpressing the DSS1(V) protein (OEX DSS1(V)). All three genotypes were cultured on solid MS media (1x MS salts, 1% sucrose, 0.05% MES, 0.7% agar, pH 5.7) (Murashige and Skoog 1962). Fourteen‐day‐old Arabidopsis seedlings were then transferred to a cultivation substrate (FloraBella) in pots and grown under long‐day conditions (14 h of light/10 h of dark) with a light intensity of 165 μmol/m^2^/s, a relative humidity of 70%, and a temperature of 21°C.

### 2.2 Generation of *DSS1(V)* Overexpressing Lines

Gateway cloning (ThermoFisher) was used to generate overexpressing lines (OE‐*DSS1(V)*). Primers attB1—GGGGACAAGTTTGTACAAAAAAGCAGGCTTAgccacc and attB2—GGGGACCACTTTGTACAAGAAAGCTGGGTA were used to amplify *DSS1(V)* CDS. The CDS was inserted into pDONR221‐P1P2 vector via BP reaction, and later on transferred into pEarlyGate100 plant expression vector—ABRC stock number CD3–724 (https://abrc.osu.edu/stocks/number/CD3‐724), via LR reaction. pEarlyGate100 vector has the strong viral 35S promoter to drive gene expression and Basta (*BlpR* gene) resistance for plant selection (Figure S2A). Mature T0 generation of *A. thaliana* Col‐0 plants was transformed with a vector carrying a T‐DNA cassette (Figure S2A) using *the Agrobacterium tumefaciens‐*mediated floral dip method (Clough and Bent 1998). T1 seedlings that carried the T‐DNA cassette were selected by spraying with Basta (Figure S2A). Two plants that survived were propagated and labeled as: OE‐*DSS1(V)0*.1 and OE‐*DSS1(V)* (Figure S2B). Primers complementary to the CaMV 35S promoter and conventional PCR were used to confirm the presence of the transgene in OE‐ *DSS1(V)0.1* and OE‐*DSS1(V)0.2* plants (Figure S2C). Plant OE‐*DSS1(V)0.2* was self‐pollinated, propagated, and used in experiments.

### 2.3 Drought Stress Treatment

After a week of growth in pots, with watering every other day, 21‐day‐old plants in three trays, each containing 24 plants, were subjected to drought conditions by withholding water, as described in Figure S1. The plants in the other three trays continued to be watered under the same regime and served as the control group. On the ninth day of the drought treatment, both treated and control plants were harvested, divided into three technical replicates, immediately transferred, and stored at −80°C for further analysis. The experiment was conducted in three biological replicates. ImageJ open‐source software was used to measure the total rosette area (Schneider et al. 2012).

### 2.4 Measurement of Relative Water Content (RWC) in Plant Tissue

Relative water content (RWC) determines the amount of water in plant tissues in relation to the state of full turgescence (complete saturation of the tissue with water). RWC was calculated using the leaf clipping method (Barrs and Weatherley 1962). The mass of fresh and dry leaf clippings was measured for three technical replicates from each of three biological experiments, conducted on different days: 0, 2, 4, 7, and 9. The area of the measured leaf clippings was 11.4 mm2. RWC was calculated according to the following formula:

$$\mathrm{RWC}\;\left(\%\right)=\frac{\mathrm{FW}-\mathrm{DW}}{\mathrm{TW}-\mathrm{DW}}\times 100$$

In the equation, RWC denotes the relative water content; FW is the fresh weight, measured immediately after harvesting the leaf sample; DW is the dry weight, obtained after drying the sample in an oven at 80°C for 24 h; and TW is the turgid weight, determined after fully hydrating the sample in distilled water overnight at 4°C in the dark. Furthermore, the water deficit (WD) in the leaves is calculated by the formula:

$$\mathrm{WD}\;\left(\%\right)=100-\mathrm{RWC}\;\left(\%\right)$$

### 2.5 Determining Substrate Water Content

The water content of the substrate was measured as previously described (Haj‐Amor et al. 2023). A small portion of the substrate was placed into pre‐weighed 2 mL microtubes, which were sealed prior to measurement to prevent evaporation. Subsequently, the substrate was transferred into measuring cups and weighed using an analytical balance. After that, the substrate was placed in a vacuum oven to dry overnight until completely dry, after which its mass was measured. The mass of the substrate was measured on the following days: 0, 2, 4, 7, and 9 from the beginning of the treatment. The obtained results are processed according to the formula:

$$\mathrm{Wd}\;\left(\%\right)=\frac{M1-M2\;}{M2-M}\times 100$$

In the formula, Wd is the moisture/water content of the substrate, M1 represents the mass of wet soil together with the container, M2 represents the mass of dry soil with the container, and M represents the mass of the weighing container.

### 2.6 Determination of Chlorophyll Content

To determine the chlorophyll content, 500 mg of previously ground plant material (prepared in liquid nitrogen) was combined with 5 mL of methanol in a 15 mL plastic test tube. The tube was wrapped in aluminum foil and placed on a rocker (Thermo Scientific) for 4 h. Afterward, the mixture was centrifuged in a cold clinical centrifuge at +4°C and 5000 rpm for 20 min. The supernatant was then transferred to a new plastic tube and wrapped in aluminum foil. The solution was diluted in methanol (950 μL of methanol and 50 μL of supernatant in a 1.5 mL microtube, 20x dilution), and its absorbance was measured at 632, 652, 665, and 696 nm using a spectrophotometer (Amersham Bioscience Ultrospec 500 pro). After obtaining the absorbance values, the total chlorophyll (Chl_tot_) content was calculated using the formula (Ritchie 2008):

$${\mathrm{Chl}}_{\mathrm{tot}}=28.6473\;A_{630}+12.9405\;A_{647}+0.6845\;A_{664}+5.2230\;A_{691}\;\left(\pm 0.0056\right)$$

The obtained values are expressed in g/L, that is, g/m^3^.

### 2.7 Determination of Total Phenols

Total phenols were measured using the Folin‐Ciocalteau method with the Folin‐Ciocalteau reagent (FC reagent) (Stanisavljević et al. 2013). The FC reagent is a combination of phosphomolybdenum and phosphotungstic acids, which, during the oxidation of phenol, are reduced to molybdenum and tungsten oxides, imparting a blue color to the final compound, which is read at 765 nm. 2 mL of 80% acetone was added to 50 mg of homogenized plant material in liquid nitrogen. The mixture was centrifuged in a cold minicentrifuge at +4°C and 10,000 rpm for 10 min. Next, 100 μL of the supernatant was collected, followed by the addition of 1500 μL of H₂O, 100 μL of FC reagent, and 300 μL of 20% Na₂CO₃. The mixture was left at room temperature for 2 h before its absorbance was measured using a spectrophotometer (Amersham Bioscience Ultrospec 500 pro). Phenol levels were determined using a standard curve based on absorbance measurements of solutions with varying concentrations of gallic acid.

### 2.8 Determination of Lipid Peroxidation Level

The level of lipid peroxidation can be determined by measuring the amount of malondialdehyde (MDA) using the TBA test (Senthilkumar et al. 2021). In this case, thiobarbituric acid (TBA) reacts with malondialdehyde (MDA), which is formed in the process of oxidative degradation of lipids. In 1.5 mL microtubes, 100 mg of plant material, previously ground in liquid nitrogen, was mixed with 800 μL of 20% trichloroacetic acid (TCA). The samples were briefly vortexed and left on ice. Centrifugation was then performed in a cold minicentrifuge at +4°C for 20 min at 13,000 rpm. After that, 600 μL of supernatant was separated into new microtubes and mixed with 600 μL of 0.5% TBA in 20% TCA. The solution was incubated in a water bath for 30 min at 95°C, quickly cooled on ice, and centrifuged in a centrifuge for 30 min at +4°C and 13,000 rpm. Then, the absorbance of the supernatant was measured at 532 nm and 600 nm on a spectrophotometer (Amersham Bioscience Ultrospec 500 pro). Lipid peroxidation is shown as MDA content per mass of plant tissue in the reaction mixture, that is, as nmol/mg and was calculated using an extinction coefficient of 155 mM–1 cm, according to the following formula:

$$\mathrm{MDA}=\frac{{А}_{532}-{А}_{600}}{155}$$

### 2.9 Detection of Protein Oxidation via OxyBlot Assay

Total proteins were isolated from *A. thaliana* ground leaves; 100 mg of plant material and 200 μL of protein extraction buffer (2.5 mM Tris–HCl, pH 7.5–8; 2 mM EDTA; 0.1 mM PMSF) were mixed. The samples were left on ice for 1.5 h, with occasional vortexing, and then centrifuged for 15 min at 10000 rpm in a cold minicentrifuge at +4°C (Eppendorf 5417R). After that, the supernatant was separated and stored until use at −20°C.

Oxidized proteins in total plant protein isolates were identified using the highly specific and sensitive OxyBlot Protein Oxidation Detection Kit (Merck Millipore, USA), which detects carbonylated groups in proteins modified by reactive oxygen species (ROS). Sample preparation followed the manufacturer's protocol. Protein extracts (~25 μg) were derivatized with 10 μL of 1 × 2–4‐dinitrophenylhydrazine (DNPH) in 6% SDS. Following incubation (15 min, RT) and neutralization, samples were stored at +4°C for up to 7 days.

Derivatized samples (see above) (10 μL) were separated via SDS‐PAGE using a 12% acrylamide gel, followed by protein transfer onto a polyvinylidene difluoride (PVDF) membrane (Immobilion‐P, Millipore) using the Fast Blot Transfer system (Biometra). After brief methanol activation, membranes were equilibrated in anode and cathode buffers, and transfer was performed for 40 min at 370 mA. Membranes were blocked with 1% bovine serum albumin (BSA) in PBST (0.1% Tween‐20 in PBS) to prevent non‐specific antibody binding. Primary antibody (1:150 in PBST) was incubated overnight at +4°C, followed by washes and incubation with a secondary antibody (1:300 in PBST) for 1 h at RT. Detection utilized the Immobilon Western Chemiluminescent HRP Substrate (Millipore), with oxidized proteins visualized using the ChemiDoc MP Imaging System (Bio‐Rad) at a 30‐s exposure. Semiquantitative analysis of the amount of oxidized proteins in relation to total proteins was performed by ImageJ software.

### 2.10 RNA Isolation and Quantitative Real‐Time PCR

Total RNA was isolated from *A. thaliana* leaf, homogenized in liquid nitrogen. The extraction was performed using the RNeasy Plant Mini Kit (Qiagen Group), following the manufacturer's instructions. The concentration of the isolated RNA was measured using a spectrophotometer (BioSpec‐nano, Shimadzu).

Prior to reverse transcription, genomic DNA contamination was removed using the DNA‐free DNA Removal Kit (Invitrogen, USA), according to a modified manufacturer's protocol. Approximately 10 μg of RNA was mixed with nuclease‐free water in 0.5 mL microtubes, reaching a final volume of 44 μL. DNase treatment was performed by adding 5 μL of 10× DNase I buffer and 1 μL of rDNase I, followed by incubation at 37°C for 30 min in a PCR machine (Professional Thermo Cycler, Biometra, Germany). DNase inactivation was achieved by adding 5 μL of DNase inactivator and incubating at room temperature for 2 min with intermittent shaking. Samples were centrifuged (Mini Spin, Eppendorf) at 10,000 × g for 1.5 min, and supernatants were transferred to new microtubes before new RNA concentration measurement. cDNA synthesis was performed using the Thermo Scientific RevertAid First Strand cDNA Synthesis Kit. Each reaction contained 1 μg of purified RNA, 1 μL of random hexamer primers (Thermo Scientific, 0.2 μg/μL), and nuclease‐free water to reach a final volume of 12.5 μL. The mixture was incubated at 65°C for 5 min and then rapidly cooled on ice for 2 min. Subsequently, 7.5 μL of master mix (comprising 4 μL of 5 × reaction buffer, 2 μL of 10 mM dNTPs, 0.5 μL of RNase inhibitor [RiboLock RNase Inhibitor, 40 U/μL], and 1 μL of reverse transcriptase [RevertAid Transcriptase, 200 U/μL]) was added. The reverse transcription reaction proceeded under the following conditions: 10 min at 25°C, 60 min at 42°C, and 10 min at 70°C, using the Professional Thermo Cycler (Biometra, Germany).

For Quantitative real‐time PCR (qRT‐PCR), cDNA samples were mixed with primers designed to amplify the gene of interest (*AthDSS1(V)*) along with an endogenous control (*AtActin*). The PCR mixture comprised 6.25 μL of 2 × SYBR Green Supermix (Applied Biosystems), 0.5 μL of primers, 4.25 μL of bidistilled water, and 1 μL of cDNA. PCR amplification was carried out using a Magnetic Induction Cycler Real‐Time PCR System under the following thermal cycling conditions: 2 min at 50°C, 10 min at 95°C, followed by 40 cycles of denaturation at 95°C for 15 s and annealing/extension at 60°C for 45 s. Actin served as an endogenous control for relative gene expression, as its expression remains highly stable across treatments, including drought (Feng et al. 2019).

### 2.11 Primer Design and Gene Expression Analysis

Primers for amplification of the RAB18 gene were designed using Primer3 software (https://primer3.ut.ee), whereas primers for amplification of RD29A were obtained from the study by Rasheed et al. 2016. The primers utilized in this study are detailed in Table 1. Relative gene expression was calculated using the 2^−ΔCt method.

**TABLE 1:** PCR primer sequences used in this study.

Gene name	Accession number	Primer name	Primer sequence	Product length (bp)
Desiccation‐responsive protein 29A	AT5G52310	RD29Af	TGGATCTGAAGAACGAATCTGATATC	45
		RD29Ar	GGTCTTCCCTTCGCCAGAA	
RAB GTPASE HOMOLOG B18	AT1G43890	RB18f	CGGGACTGAAGGCTTTGGAA	155
		RB18r	TTCCTCCTCCCTCCTTGTCC	
DSS1 homolog on chromosome V	AT5G45010	DSS1(V)f	AAGTGGTGAAGGTGGATCTATTC	193
		DSS1(V)r	CATTTCTTCTCACTAGCATTCTCAAG	
Catalase 1	AT1G20630	CAT1f	AGGAGCCAATCACAGCC	194
		CAT1r	AGGAGCCAATCACAGCC	
Glutathione synthetase 2	AT5G27380	GSH2f	ATTGGCTAAAGCTTGGTTGGAGTA	72
		GSH2r	CGTTCTTCTGGCTGTACAATTACCA	
actin‐12	AT3G46520	Actf	CTTGCACCAAGCAGCATGAA	68
		Actr	CCGATCCAGACACTGTACTTCCTT	
CaMV35S promoter		35Sf	ATTGATGTGATATCTCCACTGACGT	101
		35Sr	CCTCTCCAAATGAAATGAACTTCCT	

### 2.12 Statistical Data Processing

The collected data were statistically processed and presented as the mean value ± standard deviation (SD), which was obtained in three repeated biological experiments and two repeated measurements, within each of the applied methods. The GrafPad Prism 8.0 program was used to determine statistical significance, using Turkey's multiple comparison test through two‐way analysis of variance. In cases where the *p*‐value was less than 0.05, the differences were considered statistically significant.

## 3 Results

### 3.1 *DSS1(V)* Overexpression Lines Are Phenotypically Indistinguishable From the WT During Development

Following the characterization of knockout *dss1(V)* lines during early plant development in a prior study, the present work focuses on the phenotypic analysis of the *DSS1(V)* overexpression lines. Comparison of morphological traits revealed no visible or statistically significant differences between the OE‐*DSS1(V)* and WT plants (Figure S3). On the third day post‐germination, the average radicle length in both genotypes was 0.5 cm (Figure S3A). By the seventh day, seedling average length was 2 cm (Figure S3B). Prior to transfer onto the substrate, all seedlings measured between 5 and 6 cm (Figure S3C). Rosette areas of 24‐day‐old plants averaged 9 cm^2^ across both lines (Figure S3D). However, shoot emergence occurred earlier in OE‐*DSS1(V)* plants compared to WT, with 50% of OE‐*DSS1(V)* plants developing their first shoot by day 30, compared to 33% of WT plants. Rosette area measurements in the seventh week showed that both genotypes had an average area of 30 cm^2^ (Figure S3E). Since all three lines exhibited uniform morphology during the mid‐developmental stage of the plants, it was possible to proceed with further experiments involving stress conditions—without concern that stress would differentially affect the lines due to morphological disparities or growth delays.

### 3.2 Phenotypes of WT, KO‐*dss1(V)*, and OE‐*DSS1(V)* Lines of *Arabidopsis thaliana* Remain Unaffected by Drought Conditions

**FIGURE 1:**
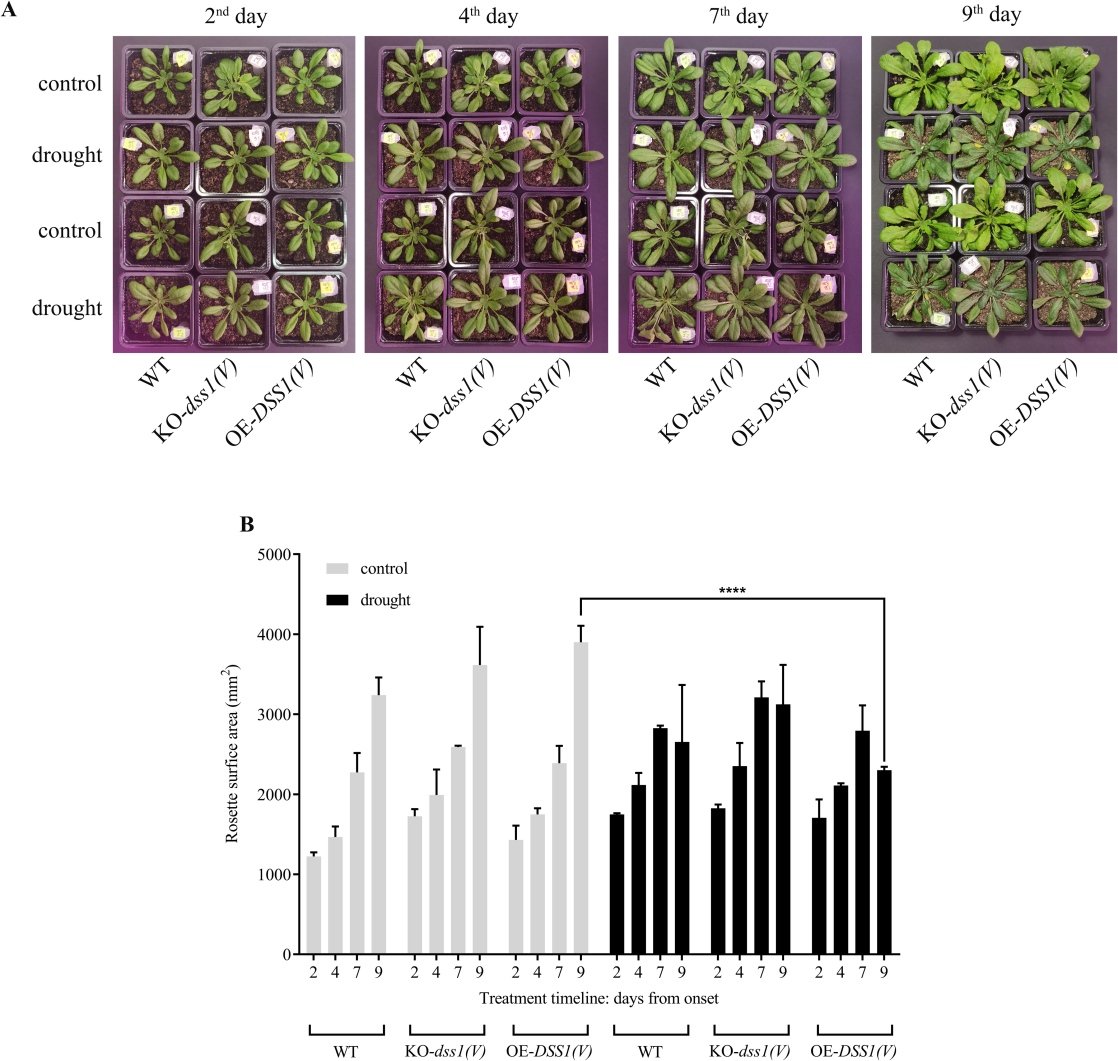
Example images of Arabidopsis plants from control and drought condition groups, 2, 4, 7, and 9 days after the start of the treatment (A) and quantification of rosette surface (B). WT, wild type plants; KO‐*dss1(V)*, *DSS(V)* knock‐out plants; OE‐*DSS1(V)*, *DSS1(V)* overexpressing plants. Data are presented as the mean±SD of values obtained from three biological experiments (*n* = 9). Significant differences in rosette size between OE‐*DSS1(V)* control and OE‐*DSS1(V)* drought‐treated samples (*p* < 0.05) are indicated by an asterisks (*).

We examined the effect of moderate drought on three lines of *A. thaliana* : WT, *dss1(V)* knockout, and DSS1(V) overexpression lines. Three‐week‐old plants were divided into control (regular watering every 2–3 days) and drought groups (no watering for 9 days). Morphological and physiological parameters were assessed after 9 days of drought treatment (Figure S1). During the early stages of the experiment, plants in both groups displayed phenotypic uniformity (Figure 1). Later on, all three genotypes under drought conditions showed reduced rosette size, gradual wilting of leaves, and a slight purple pigmentation on the dorsal side of the leaves. No significant phenotypic differences were observed among the three genotypes subjected to drought, based on visual assessment.

### 3.3 Substrate Water Content

**FIGURE 2:**
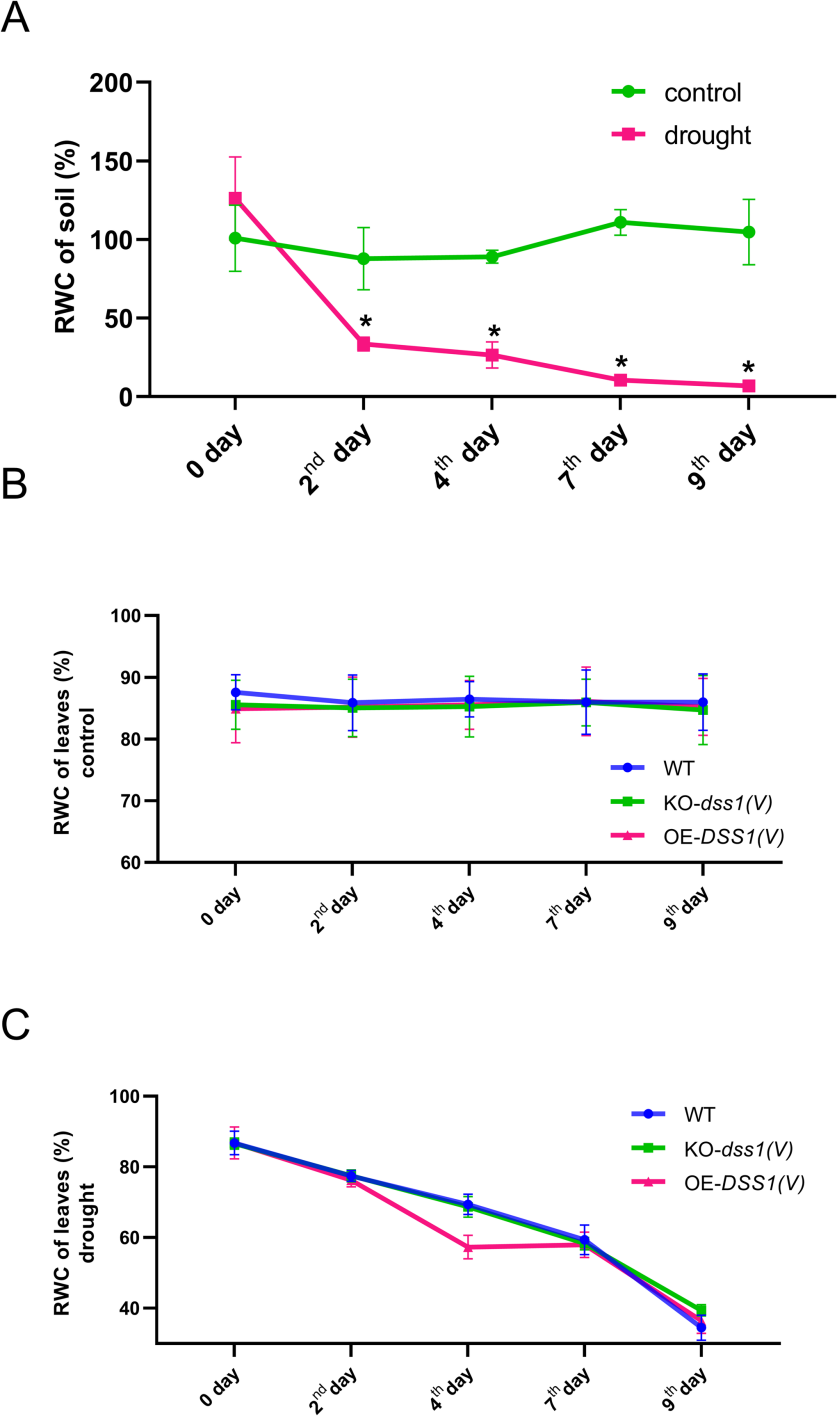
Relative water content in the soil (A) and leaves of *A. thaliana* control plants (B) and plants exposed to drought over 9 days from the onset of stress (C). WT, wild type plants; KO‐*dss1(V)*, DSS(V) knock‐out plants; OE‐*DSS1(V)*, DSS1(V) overexpressing plants. Data are presented as the mean±SD of values obtained from three biological experiments (*n* = 9). For statistical analysis, two‐way ANOVA followed by Tukey's multiple comparison test was performed using the GrafPad Prism 8.0.

To more precisely define the intensity of drought, substrate moisture was measured over time. At the start of the treatment, on day zero, substrate moisture was approximately 100% in both the control plants and the group later subjected to drought. By the second day, substrate moisture in the drought‐treated group dropped drastically to about 40%, as shown in Figure 2A, while substrate moisture in the control group remained close to the initial level. Subsequently, a gradual decline was observed, with substrate moisture reaching 10% on the seventh day and 5% on the ninth day. In contrast, substrate moisture in the control group remained stable throughout the experiment.

### 3.4 Relative Water Content (RWC) of Leaves During Drought Stress

To assess the hydration status of *A. thaliana* leaves and evaluate the severity of applied stress, relative water content (RWC) was measured. RWC quantifies the water content in leaves relative to their maximum capacity at full turgor. This parameter reflects both leaf water potential and cellular osmotic adjustment to water scarcity, facilitating comparisons among three genotypes: wild‐type plants (WT), *dss1(V)* knockout mutants (KO‐dss1(V)), and plants overexpressing the DSS1(V) protein (OE‐*DSS1(V)*). As shown in Figure 2B, RWC remained relatively stable in the control group, which was watered every other day, with minor variations between genotypes. In drought‐treated plants, RWC dropped significantly within the first 2 days across all genotypes, reaching approximately 70% (Figure 2C). By the fourth day, RWC declined further to ~65% in WT and mutant plants, while OEV plants exhibited a more pronounced reduction to ~56%. This trend continued, with all genotypes showing a proportional decrease in RWC on the seventh and ninth days of drought. By the ninth day, leaves across all genotypes appeared severely desiccated, with RWC levels measuring just above 30%.

### 3.5 Total Chlorophyll Content

**FIGURE 3:**
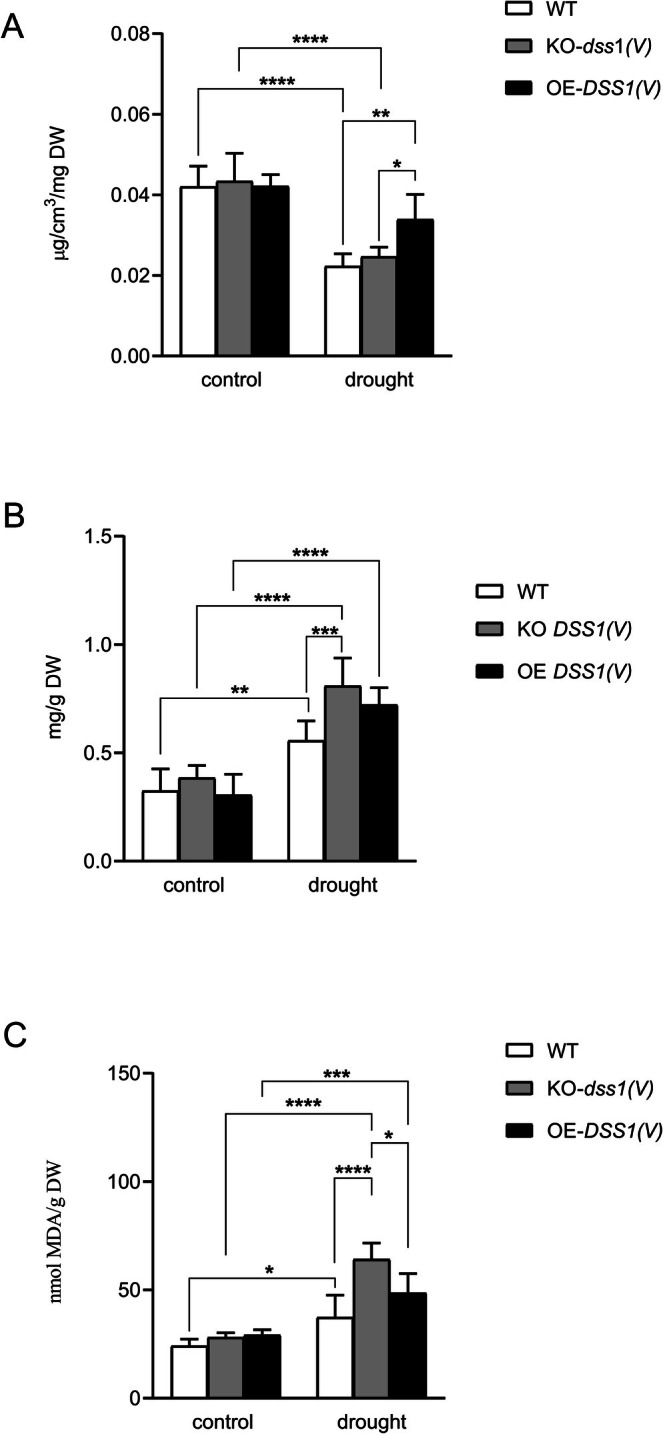
Graphical representation of the levels of total chlorophyll (A), total phenols (B), and lipid peroxidation (C) of *A. thaliana* control plants and plants exposed to drought over 9 days. Data are presented as the mean ± SD of values obtained from three biological experiments (*n* = 9). Significant differences (*p* < 0.05) between samples are indicated by *. For statistical analysis, two‐way ANOVA followed by Tukey's multiple comparison test was performed using the GrafPad Prism 8.0.

The content of plant pigments is closely linked to overall plant fitness and stress resistance. Chlorophyll content, in particular, serves as a sensitive indicator of abiotic stress, as it undergoes significant changes under such conditions (Kalaji et al. 2016). During drought, stomatal closure reduces water loss but also limits CO₂ uptake, leading to disruptions in chlorophyll synthesis (Collin et al. 2025). Additionally, drought conditions accelerate chlorophyll degradation and leaf yellowing, directly impacting the plant's ability to convert light energy and CO₂ into organic compounds and O₂. As such, we quantified the total chlorophyll content in leaf dry mass of the control group and drought‐treated plants of three genotypes (Figure 3A). Total chlorophyll content was significantly higher in plants grown under control conditions compared with those subjected to drought. Within the control group, no significant differences in chlorophyll content were detected among genotypes. Under drought conditions, the OE‐*DSS1(V)* plants had the highest chlorophyll content among the three genotypes, suggesting a reduced stress load in that background.

### 3.6 Content of Total Phenols

Phenolic compounds play a crucial role in plants, particularly under stress conditions. They are an integral part of the plant defense system against biotic stressors such as pathogens or herbivores, as they can inhibit pathogen growth and deter herbivores (Kumar et al. 2023). Additionally, phenolics possess strong antioxidant properties, aiding in the elimination of reactive oxygen species (ROS) generated during abiotic stresses such as drought, high salinity, or extreme temperatures (Kumar et al. 2023). Phenolics are also involved in signaling pathways that activate stress response mechanisms, modulate the expression of stress‐responsive genes, and regulate various physiological processes, including growth, development, and reproduction (Kumar et al. 2023). The phenolic levels in plants are an important stress parameter, so we quantified phenolic content in control plants and those subjected to drought (Figure 3B). The total phenolic content was higher in plants of all three genotypes grown under drought conditions compared to the control group. Within the control group, no significant differences were observed between genotypes. However, in the drought‐treated group, KO‐*dss1(V)* plants have significantly higher phenolic levels compared with the WT. Quantification of phenolic content revealed that drought conditions increased phenolics compared to the control by approximately 1.8‐fold in WT plants, while a more pronounced increase of 2.2 and 2.4‐fold was observed in OE‐*DSS1(V)* and KO‐*dss1(V)* plants, respectively. The total phenolic content, as a stress indicator, suggests that knockout *dss1(V)* plants were the most affected by drought.

### 3.7 Lipid Peroxidation

Lipid peroxidation (LPO) refers to the oxidative degradation of lipids, leading to the formation of reactive aldehydes, including malondialdehyde (MDA). As a chain reaction, LPO causes significant damage to cell membranes and other lipid‐containing structures (Ayala et al. 2014). MDA, a final product of polyunsaturated fatty acid peroxidation, is widely used as a biomarker for oxidative stress, with elevated levels indicating increased LPO and oxidative damage (Cordiano et al. 2023). To assess the stress response in plants, malondialdehyde levels were measured (Figure 3C). LPO levels were significantly higher in drought‐treated plants across all genotypes compared to the control group, in which no significant differences between genotypes were observed. Under drought conditions, KO‐*dss1(V)* plants exhibited significantly higher LPO levels compared with the WT and OE‐*DSS1(V)* plants. Specifically, LPO levels in KO‐*dss1(V)* plants were 2.28 times higher than in the controls, while WT and OE‐*DSS1(V)* plants showed increases of 1.55 and 1.66 times, respectively. These findings indicate that the KO‐*dss1(V)* genotype is more sensitive to drought than the other genotypes.

### 3.8 Protein Oxidation

**FIGURE 4:**
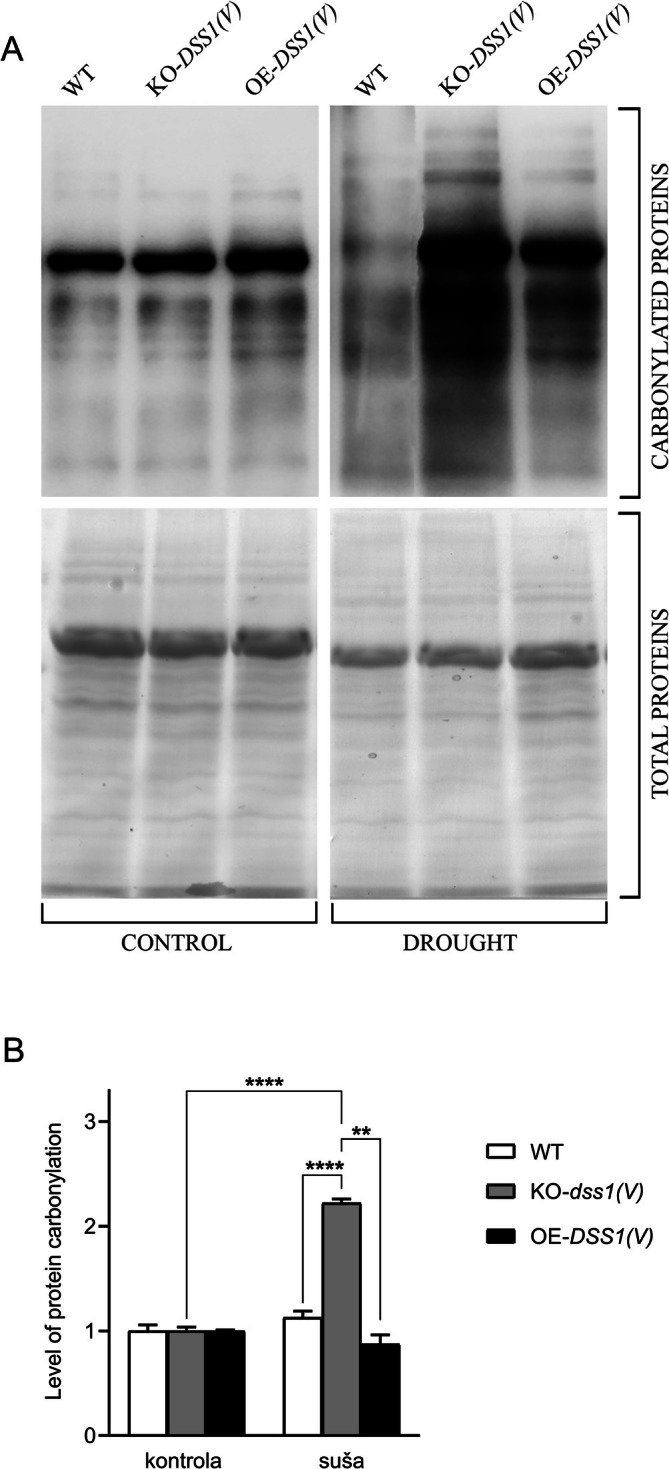
Determination of protein carbonylation: (A) OxyBlot‐ (top) and Coomassie‐Brilliant Blue (CBB) stained SDS‐PAGE (bottom) and (B) Level of protein carbonylation (total band densities were determined by ImageJ software for densitometry. Data shown are representative of three experiments; relative band density is presented as the total band density of carbonylated proteins detected by anti‐DNP antibodies/band density of total proteins stained with CBB. Experiments were performed in biological triplicates and data correspond to the mean ± SD. *p < 0.05 indicate a statistically significant difference.

Oxidative stress or accumulation of reactive oxygen species (ROS) induced by various biotic and abiotic stresses such as drought lead to irreversible protein carbonylation (Møller et al. 2007). Carbonyl groups (aldehydes and ketones) form on protein side chains and serve as reliable markers of oxidative damage (Dalle‐Donne et al. 2003). The levels of carbonylated proteins in total protein extracts from WT, KO*‐dss*1*(V)*, and OE‐*DSS1(V)* plants, grown under normal physiological conditions or drought, were quantified using the immunoblot method. Proteins were first derivatized with 2,4‐dinitrophenylhydrazine, which reacts with carbonyl groups to produce DNP‐hydrazone. These DNP‐modified proteins were subsequently detected using antibodies and quantified densitometrically using Scion Image software. The results shown in Figure 4, indicate that under drought conditions, KO‐*dss1(V)* plants exhibited a markedly higher level of carbonylated proteins compared to WT and OE‐*DSS1(V)* plants, highlighting the increased sensitivity of the mutant genotype to drought stress. Within the control group, no significant differences in carbonylated protein levels were observed among genotypes.

### 3.9 Analysis of *DSS1(V)*, *RD29A*, *RAB18*, *CAT1*, and *GSH2* Gene Expression

**FIGURE 5:**
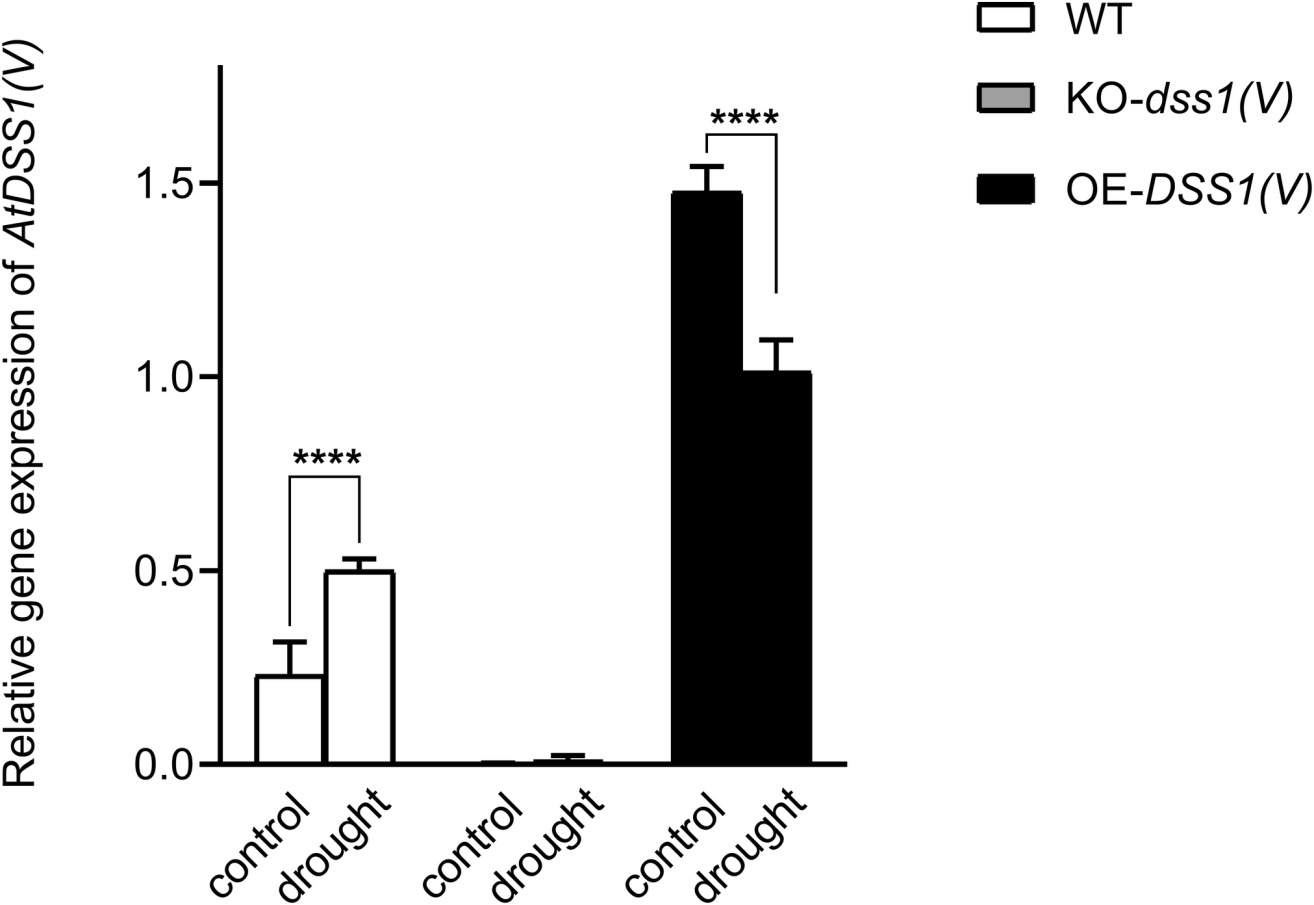
Relative expression of the *DSS1(V)* gene in three *A. thaliana* genotypes grown under normal and drought conditions. Gene expression is determined using the 2^–ΔΔCt^ method. Asterisks on the graph indicate the existence of a statistically significant difference based on Tukey's multiple comparison test (*p* ≤ 0.05).

To confirm the role of the small, intrinsically disordered protein DSS1(V) in plant responses to drought stress, the level of *DSS1(V)* transcripts was monitored in different *A. thaliana* genotypes grown under normal physiological conditions or exposed to drought using the qPCR method. We examined *DSS1(V)* transcript levels in WT plants to assess its drought stress responsiveness, while also verifying gene deactivation and overexpression in the respective mutant lines. The results (Figure 5) showed that WT plants exposed to drought conditions exhibited a statistically significant increase in *DSS1(V)* transcript levels compared to those grown under normal conditions. At the same time, we confirmed the absence of the *DSS1(V)* transcript in knockout plants, as expected, due to the targeted disruption of this gene using CRISPR/Cas9 technology. In OE‐*DSS1(V)* lines, both control and drought‐exposed plants exhibited elevated *DSS1(V)* transcript levels, 6.43 fold and 4.39 fold, respectively, compared with the control WT plants. Surprisingly, the OE‐*DSS1(V)* plants, which use a strong viral 35S promoter, exposed to drought had lower *DSS1(V)* compared to the control group, suggesting a post‐translational regulation of the transcript.

We also analyzed the expression of selected genes known to undergo significant changes during drought stress, such as *RAB18* and *RD29* (*Responsive to ABA 18* and *Responsive to Desiccation 29A*) (Harb et al. 2010; Shinozaki and Yamaguchi‐Shinozaki 2007), as well as genes that exhibit moderate responsiveness to other stress conditions, including *CAT1* (*Catalase* 1) and *GSH2* (*Glutathione Synthetase 2*), which can be considered stress markers (Xie et al. 2012; Uzilday et al. 2018). We quantified transcript levels of these marker genes in all three genotypes cultured under both control and drought conditions.

**FIGURE 6:**
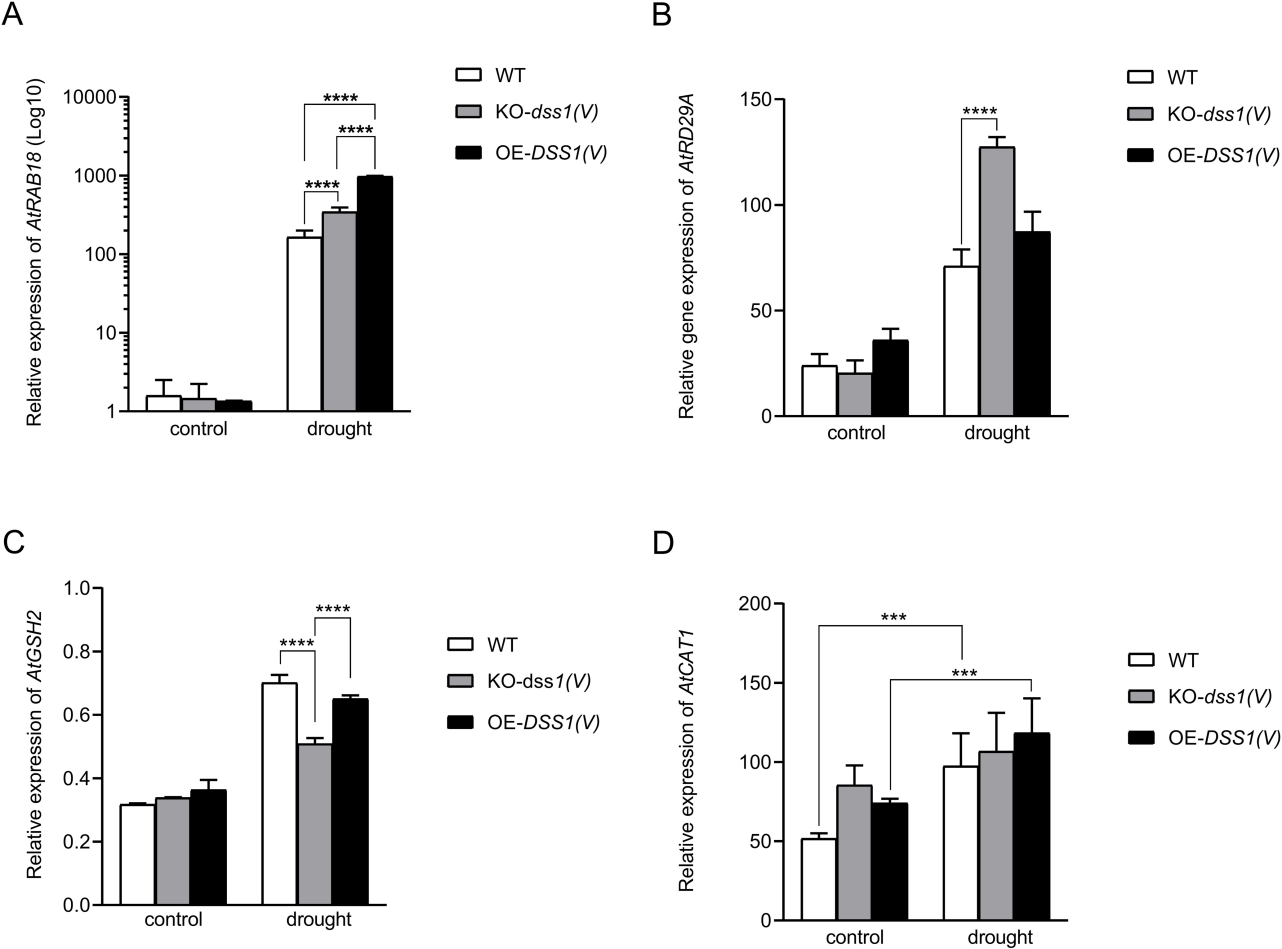
Relative expression of the *RD29A* (A), *RAB18* (B), *GSH2* (C), and *CAT1* (D) genes in three *A. thaliana* genotypes grown under normal and drought conditions. Gene expression is determined using the 2^–ΔΔCt^ method. Asterisks on the graph indicate the existence of a statistically significant difference based on Tukey's multiple comparison test (*p* ≤ 0.05).

*RAB18* gene encodes a GTPase‐functioning protein and is part of the dehydrin family (Nylander et al. 2001). Arabidopsis dehydrin RAB18 is upregulated in response to drought, freezing, osmotic stress, ABA, etc. (Nylander et al. 2001) and hence serves as a strong stress indicator. We found that *RAB18* transcript levels increased across all three genotypes under drought conditions: WT plants exhibited a 103‐fold increase, KO‐*dss1(V)* plants a 238‐fold increase, and OE‐*DSS1(V)* plants a dramatic 752‐fold increase compared to their respective controls (Figure 6A). We also found a statistically significant difference in *RAB18* expression among all genotypes grown under drought conditions, with OE‐*DSS1(V)* plants displaying the highest expression levels in response to drought. This suggests that overexpression of DSS1(V) might trigger an alternative stress response under drought conditions.

The *RD29A* gene is highly responsive to abiotic stress, particularly drought, cold, and high salinity (Jia et al. 2012). Compared to control plants, *RD29A* transcript levels were 3.1 times higher in WT plants, 6.35 times higher in KO plants, and 2.4 times higher in OE plants when exposed to drought (Figure 6B). Among the genotypes, KO plants exhibited the highest increase in expression levels under drought, with 1.8‐fold and 1.4‐fold higher expression than WT plants and OE plants under drought, respectively (Figure 6B).

We also tested the expression of two additional genes known to be responsive to drought stress—*catalase 1* (*CAT1*) and *glutathione synthase 2* (*GSH2*) (Figure 6C,D). *CAT1* transcript levels under drought conditions were elevated in both WT and OE genotypes compared to the control, by 1.9‐fold and 1.2‐fold, respectively (Figure 6C). However, unlike WT and OE‐*DSS1(V)* plants, *CAT1* expression in the KO line did not show a statistically significant increase under drought compared to the control. On the other hand, *GSH2* gene expression was significantly elevated across all genotypes, with the most substantial increase observed in WT plants exposed to drought. These plants exhibited *GSH2* transcript levels 2.2 times higher than WT control plants (Figure 6D). In drought conditions, KO plants showed a more moderate increase in *GSH2* expression, with transcript levels approximately 1.3 times higher than in control plants.

## 4 Discussion

Plants, as sessile organisms, face biotic and abiotic stress, which has intensified with climate change. Through evolution, they have developed sophisticated defense mechanisms, including active biomolecules that degrade cellular components damaged by oxidative stress (Mareri et al. 2022). Oxidized proteins formed during oxidative stress are often toxic, prompting rapid elimination. A recent study identified DSS1 proteins as key players in tagging these proteins for degradation, in a process called DSSylation (Zhang et al. 2014). While the exact mechanism remains unclear, this discovery highlights DSS1's role in post‐translational protein modification.

Based on our previous findings (I. P. Nikolić et al. 2021; I. Nikolić et al. 2023), we hypothesized that overexpression of DSS1 proteins could enhance plant resistance to drought‐induced oxidative stress. Excess DSS1 proteins may facilitate more efficient elimination of damaged proteins, activate transcription of defense genes, or provide mechanical protection by forming a “molecular shield.” Conversely, disruption of the *DSS1* gene may increase plant sensitivity to oxidative stress for the same reasons. Since prior research (I. P. Nikolić et al. 2021) indicated that the *DSS1(V)* gene responds strongly to stress induced by hydrogen peroxide and methyl viologen, it was chosen for further analysis for its role in drought stress response, as drought leads to extensive production of ROS molecules, making oxidative stress a significant secondary consequence.

It is important to note that various methods exist to induce a water deficit in potted plants. Despite extensive research on drought effects, there is still no consensus on the best approach for simulating drought under experimental conditions (Munns 2011). The simplest method—passive drying by interrupting irrigation—can lead to rapid drying, which does not accurately replicate natural soil water deficits (Poorter et al. 2011). Some studies have used osmotically active substances, such as polyethylene glycol (PEG), to induce drought (Zur 1966). However, PEG alters osmotic potential, affecting water uptake, oxygen diffusion in roots (Mexal et al. 1975), and ion absorption (Yeo and Flowers 1984), causing a more complex stress than water deficit alone. Other approaches include reducing water pressure in microporous tubes (Steinberg and Henninger 1997) or using vacuum pumps on pots (Bunce and Nasyrov 2012), but both require additional equipment and logistical complexity. The most widely used method is air drying, where individual pots are regularly weighed, and precise amounts of water are added to balance transpiration losses and maintain target soil moisture levels (Earl 2003). While effective in simulating natural drought for plants of various sizes, this technique often relies on an expensive automated system. Manual implementation is possible but labor‐intensive, especially for large‐scale experiments. We all these conciderations, we chose to stimulate drought by omitting irrigation for nine days, while we kept the air humidity constant.

We confirmed the adequacy and intensity of our stress simulation by monitoring soil water content over nine days (Figure 2A). Measurements showed a progressive decrease in substrate humidity in the drought group, as expected, while the control group exhibited minimal, statistically insignificant differences. According to literature, substrate humidity between 80% and 100% does not induce stress, 40%–60% causes moderate stress, and below 40% leads to severe physiological disturbances in most plants (Laxa et al. 2019). In our drought induction system, by the ninth day—when plant material was sampled for analysis—soil water content had declined to around 5%, indicating intense stress for the plants.

The effects of drought on plant growth and development have been extensively studied (Farooq et al. 2009). While water deficit impacts numerous plant functions, its effects depend on stress intensity, duration, and plant genetics (Seleiman et al. 2021). Research has shown that drought stress reduces growth, affecting leaf area, dry weight, and other developmental factors (Fischer 1980). Relative water content (RWC) in leaves is commonly used to monitor drought effects and determine tolerance (Nxele et al. 2017). Our results showed a significant decrease in RWC across all drought‐treated plant groups compared to the control (Figure 2B,C). For the first 4 days, RWC values suggest moderate stress, while stress becomes more severe by the seventh and ninth days, with RWC dropping to around 30%. Visual observations revealed no major differences between genotypes under drought conditions; however, all showed a reduction in leaf area compared to the control after 9 days of treatment (Figure 1). Reduced leaf area under water deficit is believed to result from a slower cell elongation rate, leading to smaller cell size (Schuppler et al. 1998). This response is linked to increased abscisic acid synthesis, which inhibits the proton pump on the plasma membrane, preventing cell wall acidification and elongation (Emenecker and Strader 2020). Consequently, the expression of genes for proline‐rich protein synthesis increases, potentially restricting wall extensibility (Zdanio et al. 2020).

Furthermore, we found a clear disturbance in cellular balance upon drought stress, as indicated by increased lipid peroxidation levels (Figure 3C). Numerous studies confirm that lipid peroxidation increases under stress, leading to a rise in ROS production (Wang et al. 2023). Polyunsaturated fatty acids (PUFA), key components of biomembranes, are highly susceptible to peroxidation under adverse conditions (Yamauchi et al. 2008). This process generates various reactive compounds, including aldehydes, alkanes, alcohols, and epoxy lipids (Esterbauer et al. 1991). Malondialdehyde (MDA), one of the most reactive aldehyde forms, is produced through PUFA oxidation and interacts with biomolecules like proteins (Esterbauer et al. 1991; Yamauchi et al. 2008). In our experiments, MDA served as an indicator of oxidative stress in *A. thaliana* leaf tissues. Lipid breakdown products react with thiobarbituric acid (TBA) (Abeyrathne et al. 2021), forming thiobarbituric acid reactive substances (TBARS), which we used to quantify MDA levels as an indicator of lipid peroxidation intensity. All genotypes grown under drought showed increased lipid peroxidation, with the KO‐*dss1(V)* genotype exhibiting the highest increase (Figure 3C), indicating greater sensitivity to this stress compared to other genotypes. These findings highlight DSS1(V) as a crucial factor in *A. thaliana* 's response to oxidative stress.

The negative effect of drought on Arabidopsis plants was also evident from the total phenol content in leaf tissues. Plant phenols, a group of secondary metabolites, play a crucial role in responding to biotic and abiotic stressors (Dai and Mumper 2010). Plants adapt to environmental changes through various mechanisms that recognize and counteract stress, including drought (Hura et al. 2008). Zahedi et al. 2024 found that phenols are essential for ROS removal during dehydration. In agreement with this, our results showed a significant increase in total phenol content across all drought‐exposed genotypes, with the highest levels in the KO‐*dss1(V)* mutant (Figure 3B). The elevated phenolic content in the *dss1(V)*‐knockout mutant suggests increased stress levels, likely due to impaired oxidized protein elimination. The visual observations of drought‐grown plants (Figure 1) revealed changes in leaf color on the reverse side of the leaves, supporting the measured elevated phenol concentration (Sarker and Oba 2018). The rise in phenols in drought‐affected plants is likely a defense mechanism, as phenolic compounds serve as filters protecting the photosynthetic apparatus and function as potent antioxidants (Hura et al. 2008). Their increased synthesis during drought likely contributes to plant stress resistance (Hura et al. 2008).

As mentioned earlier, drought impacts various metabolic and physiological processes in plants, including photosynthesis. Mafakheri et al. 2010 found that drought conditions reduce stomatal conductance, directly affecting CO_2_ fixation and photosynthesis. Water deficit also influences photosynthesis by altering chlorophyll content and damaging the photosynthetic apparatus (Urban et al. 2017). In agreement, our total chlorophyll content analysis showed that WT and KO‐*dss1(V)* plants grown in drought had reduced chlorophyll content (Figure 3A). Smirnoff (1995) established that chlorophyll decline during drought stress results from oxidative damage to chloroplasts. However, we also found that plants overexpressing DSS1(V) did not show a statistically significant decrease in chlorophyll levels compared to OE control plants. This suggests that, when overexpressed, DSS1(V) may play a role in protecting the photosynthetic apparatus. However, it is worth noting that no other stress parameters indicated greater stress resistance in OE plants compared to WT. This raises the possibility that DSS1(V) has a specific protective role in preventing chlorophyll degradation during drought.

We also estimated the accumulation of proteins damaged by oxidative stress in drought‐exposed plants as an indicator of stress (Figure 4). Oxidized proteins can impair essential cellular functions by inhibiting enzymes, disrupting metabolic pathways, and damaging cellular structures (Sitte 2003). Immunoblot detection of carbonylated groups revealed the highest percentage of oxidized proteins in KO‐*dss1(V)* plants under drought—1.6 times higher than in WT plants and 2.5 times higher than in OE‐*DSS1(V)* plants in the same conditions. These findings highlight the DSS1(V) gene's crucial role in detoxifying cells from oxidative stress‐damaged proteins and maintaining protein homeostasis (I. P. Nikolić et al. 2021). DSS1(V) likely contributes to proteostasis by stabilizing the 26S proteasome architecture and enhancing its efficiency in removing oxidized proteins (Paraskevopoulos et al. 2014).

To test the level of drought stress response in various Arabidopsis lines we quantified transcriptional levels of *RAB18* and *RD29A*, two marker genes for drought stress (Harb et al. 2010; Shinozaki and Yamaguchi‐Shinozaki 2007). Drought exposure consistently induced strong expression of the RAB18 and RD29A genes across genotypes (Figure 6A,B). RD29A is expressed in response to desiccation and its promoter has dehydration responsive elements (Jia et al. 2012). Our results showed that RD29A transcript levels increased in all drought‐exposed genotypes compared to controls. The highest RD29A expression was observed in KO‐*dss1(v)* plants under drought, suggesting that those plants are experiencing heightened stress levels and need more protective proteins, like RD29A. In this context, the heightened stress sensitivity observed in the mutant may stem from a compromised capacity to detoxify oxidized proteins.

Notably, OE‐*DSS1* lines exhibited markedly elevated RAB18 transcript levels compared to mutant counterparts. One of the assumptions is that DSS1(V) overexpression may impose an additional layer of physiological stress, potentially disrupting tightly regulated gene networks and thereby triggering alternative stress response pathways. Importantly, the heightened expression of this stress‐associated gene does not necessarily reflect increased sensitivity to stress (Panjabi‐Sabharwal et al. 2009). Rather, stress markers such as RAB18 can also indicate the activation of protective mechanisms aimed at enhancing stress tolerance. Further, we hypothesize that DSS1(V) overexpression may influence abscisic acid (ABA) signaling, a key regulatory pathway in drought adaptation. ABA is known to activate transcription factors that drive the expression of dehydrin genes, including RAB18, thereby amplifying secondary stress responses and promoting cellular protection. In addition, it remains plausible that DSS1(V) functions as a “universal molecular clip,” stabilizing components of the transcriptional machinery involved in RAB18 regulation. Such stabilization could facilitate more efficient gene expression, contributing to enhanced stress resilience under drought conditions.

It was found that transcript levels of CAT1 and GSH2 were elevated in WT and OE genotypes under drought conditions (Figure 6C,D), as expected (Xie et al. 2012; Uzilday et al. 2018). However, in the KO line, CAT1 expression did not show a statistically significant increase under drought compared to the control, unlike in WT and OE‐*DSS1(V)* lines. Additionally, GSH2 expression in the KO line was significantly lower under drought compared to WT and OE lines. The underlying reason for the consistently low or unchanged transcript levels of these stress‐related parameters in mutant lines remains to be elucidated. One plausible explanation is that DSS1 plays a role in the activation of these genes, and its disruption may have impaired the proper initiation of stress‐responsive pathways. In WT plants, catalase (CAT1) plays a key role in detoxification, neutralizing H_2_O_2_ through decomposition (Xie et al. 2012), while GSH2 glutathione synthetase (GSH2) catalyzes ATP‐dependent GSH synthesis from gamma‐glutamylcysteine and glycine (Uzilday et al. 2018). As an antioxidant, glutathione protects cellular components from damage caused by reactive oxygen species such as free radicals and peroxides (Szalai et al. 2009). DSS1(V) may hypothetically play a role in transcriptional regulation (I. Nikolić et al. 2023) by stabilizing interactions within the transcription complex or enhancing its connection with DNA, thus influencing the expression of CAT1 and GSH2 under drought conditions.

Overall, it is noteworthy that our results show no significant differences in leaf relative water content (RWC) detected among the analyzed genotypes under drought stress. In contrast, obvious differences emerged in stress‐associated physiological parameters and in the expression profiles of stress‐responsive genes. Previous studies have shown that RWC is recognized as a relatively coarse physiological indicator of drought, typically showing measurable changes only at more advanced stages of water deficit (Akter et al. 2023). On the other hand, our findings reveal subtle yet informative differences in earlier and more sensitive stress markers between mutant and WT plants. The absence of significant variation in RWC across genotypes, despite clear differences in parameters such as chlorophyll content, lipid peroxidation, total phenolic accumulation, and levels of oxidized proteins, suggests that DSS1(V) may play a direct and specific role in modulating stress responses. This involvement is likely mediated through participation in distinct signaling pathways or via transcriptional regulation of selected stress‐responsive genes.

Alongside this, we examined *DSS1(V)* transcript levels to assess its response to drought stress in wild‐type (WT) plants (Figure 5). The observed upregulation of *DSS1(V)* in wild‐type (WT) plants indicates its responsiveness to stress. This aligns with our previous findings, where *DSS1(V)* was also upregulated upon exposure to hydrogen peroxide and methyl viologen, confirming its role in oxidative stress regulation (I. Nikolić et al. 2023; I. P. Nikolić et al. 2021). Since oxidative stress is a secondary effect of drought, DSS1(V) may interact with oxidized proteins, marking them for degradation, thus influencing gene expression (I. P. Nikolić et al. 2021). All together, these findings underscore DSS1(V) as a potentially significant factor in cellular adaptation to oxidative stress Somewhat surprisingly, we found that, in OE‐*DSS1(V)* plants under drought, the *DSS1(V)* transcript levels were lower compared with the control. This could be a consequence of 35S promoter expression variability. Kiselev et al. (2021) demonstrated that transgenes driven by constitutive promoters like 35S can exhibit transcriptional variability depending on plant organ, physiological state, and abiotic stress responses. Additionally, Boyko et al. (2009) found that exposure to UVB, UVC, and X‐radiation reduced 35S‐driven transgene expression in tobacco, while high temperatures increased transcription. However, we also cannot exclude other possible explanations for the observed transcript reduction.

Altogether, our data indicate that DSS1(V) may be a relevant factor in the oxidative stress response of *Arabidopsis thaliana* leaves. Although it is not an essential component, as the gene deletion is not lethal, it nevertheless significantly contributes to the efficient functioning of the proteasome machinery, which is responsible for preserving cellular homeostasis. These results should encourage further research into the functional properties of this gene in response to oxidative stress.

## Author Contributions

Gordana Timotijević and Ivana Nikolić contributed to the idea and experimental design and carried out experimental work. Ivan Radin constructed pEarleyGate100 vector carrying *DSS1(V)* gene for its overexpression. Maja Sabljić conducted water deficit experiments and measurements of stress parameters. Data analysis and the first draft of the manuscript were written by Gordana Timotijević. Ivana Nikolić, Ivan Radin, and Mira Milisavljević provided critical feedback and analysis of the manuscript. All authors read and approved the final manuscript. Language editing assistance was provided using ChatGPT (OpenAI), a large language model‐based tool. The tool was used solely to improve grammar, clarity, and fluency of the manuscript text. No content was generated by AI, and all scientific ideas, interpretations, and conclusions are the authors' own.

## Funding

This work was supported by the Science Fund of the Republic of Serbia, Grant No. 7730230, “Factors of the BRCA2‐mediated Homologous Recombination: Uncovering New Play‐ers, their Interplay, and Contribution to Genome Integrity and Stress Response”—GENOVA and Grant No. 6473515 “Overexpression of Intrinsically Disordered Protein AtDSS1 in different plant systems – an analysis of its role in response to the oxidative stress” – OXINDISPRO as well as by the Fund of the Ministry of Science, Technological Development and Innovations of the Republic of Serbia, registration number: 451‐03‐136/2025‐03/200042 agreement on the implementation and financing of research in 2025.

## Conflicts of Interest

The authors declare no conflicts of interest.

## Supporting information


**Figure S1:** Schematic representation of the experimental setup for drought treatment.
**Figure S2.** Strategy for Generating Individual *A. thaliana* Lines with Enhanced Expression of *DSS1(V)*. **(A)** Schematic representation of the T‐DNA insert in the binary vector pEarlyGate 100‐*DSS1(V)*: RB/LB—border sequences of the T‐DNA insert; MASt—mannopine synthase terminator; BlpR—gene conferring resistance to Basta herbicide; MASp—mannopine synthase promoter; CaMV 35Sp—strong constitutive promoter from cauliflower mosaic virus 35S; attL1/attL2—recombination sites for the Gateway BP reaction; *DSS1(V)*—cDNA sequence of the gene of interest; OCSt—octopine synthase terminator. **(B) T**wo transgenic plants OE *DSS1(V)0.1*, and OE *DSS1(V)0.2*, acquired following Basta treatment. **(C)** Detection of CaMV 35S promoter presence using PCR with primers 35Sf/35Sr on genomic DNA (gDNA) samples from WT plants and OE *DSS1(V)0.1 and* OE *DSS1(V)0.2* (labeled OE(V)0.1 and OE(V)0.2 in the figure).
**Figure S3:** Comparative Analysis of the Phenotype of WT and OE DSS1(V) A. thaliana Lines During Development. After two weeks of germination and growth on MS medium in Petri dishes, seedlings were transfered into soil. (A) Photographs of three‐day‐old WT and OE DSS1(V) seedlings in Petri dishes; the histogram represents radicle length in cm. (B) Photographs of seven‐day‐old seedlings in Petri dishes; the histogram represents seedling length in cm. (C) Photographs of 14‐day‐old seedlings in Petri dishes; the histogram represents seedling length in cm. (D) Photographs of 24‐day‐old seedlings in soil; the histogram represents rosette area in cm2. (E) Photographs of seven‐week‐old plants in soil; the histogram represents rosette area in cm2. White bars correspond to measurements of various parameters in WT plants, while black bars represent OE DSS1(V) plants. Representative phenotypes of the plants are shown. Results are presented as mean ± SD, obtained from three independent biological replicates (n = 20 per experiment).

## Data Availability

The data that support the findings of this study are available from the corresponding author upon reasonable request.
