# Supplementary material for: The Arabidopsis Mutant of the Small Intrinsically Disordered Protein DSS1(V) Exhibits Increased Sensitivity to Drought Stress
Source: Plant Direct. 2026 Feb 5;10(2):e70140. doi: 10.1002/pld3.70140 (PMC12873854; doi:10.1002/pld3.70140)
Supplement: Supplementary file 1 — Figure S1: Schematic representation of the experimental setup for drought treatment. Figure S2. Strategy for Generating Individual A. thaliana Lines with Enhanced Expression of DSS1(V). (A) Schematic representation of the T‐DNA insert in the binary vector pEarlyGate 100‐DSS1(V): RB/LB—border sequences of the T‐DNA insert; MASt—mannopine synthase terminator; BlpR—gene conferring resistance to Basta herbicide; MASp—mannopine synthase promoter; CaMV 35Sp—strong constitutive promoter from cauliflower mosaic virus 35S; attL1/attL2—recombination sites for the Gateway BP reaction; DSS1(V)—cDNA sequence of the gene of interest; OCSt—octopine synthase terminator. (B) Two transgenic plants OE DSS1(V)0.1, and OE DSS1(V)0.2, acquired following Basta treatment. (C) Detection of CaMV 35S promoter presence using PCR with primers 35Sf/35Sr on genomic DNA (gDNA) samples from WT plants and OE DSS1(V)0.1 and OE DSS1(V)0.2 (labeled OE(V)0.1 and OE(V)0.2 in the figure). Figure S3: Comparative Analysis of the Phenotype of WT and OE DSS1(V) A. thaliana Lines During Development. After two weeks of germination and growth on MS medium in Petri dishes, seedlings were transfered into soil. (A) Photographs of three‐day‐old WT and OE DSS1(V) seedlings in Petri dishes; the histogram represents radicle length in cm. (B) Photographs of seven‐day‐old seedlings in Petri dishes; the histogram represents seedling length in cm. (C) Photographs of 14‐day‐old seedlings in Petri dishes; the histogram represents seedling length in cm. (D) Photographs of 24‐day‐old seedlings in soil; the histogram represents rosette area in cm2. (E) Photographs of seven‐week‐old plants in soil; the histogram represents rosette area in cm2. White bars correspond to measurements of various parameters in WT plants, while black bars represent OE DSS1(V) plants. Representative phenotypes of the plants are shown. Results are presented as mean ± SD, obtained from three independent biological replicates (n = 20 per experiment). [file PLD3-10-e70140-s001.docx]

**Supplementary material**

**The Arabidopsis Mutant of the Small Intrinsically Disordered Protein DSS1(V) Exhibits Increased Sensitivity to Drought Stress**

Ivana Nikolić^1^, Maja Sabljić^2^, Mira Milisavljević^1^, Ivan Radin^3^,Gordana Timotijević^1^*

^1^Institute of Molecular Genetics and Genetic Engineering, University of Belgrade, Group for Plant Molecular Biology, Vojvode Stepe 444a, 11055 Belgrade, Serbia

^2^Belgrade, Serbia

^3^Department of Plant and Microbial Biology, University of Minnesota, St. Paul, MN 55108, USA

**Corresponding author e-mail:** [**timotijevic@imgge.bg.ac.rs**](mailto:timotijevic@imgge.bg.ac.rs)


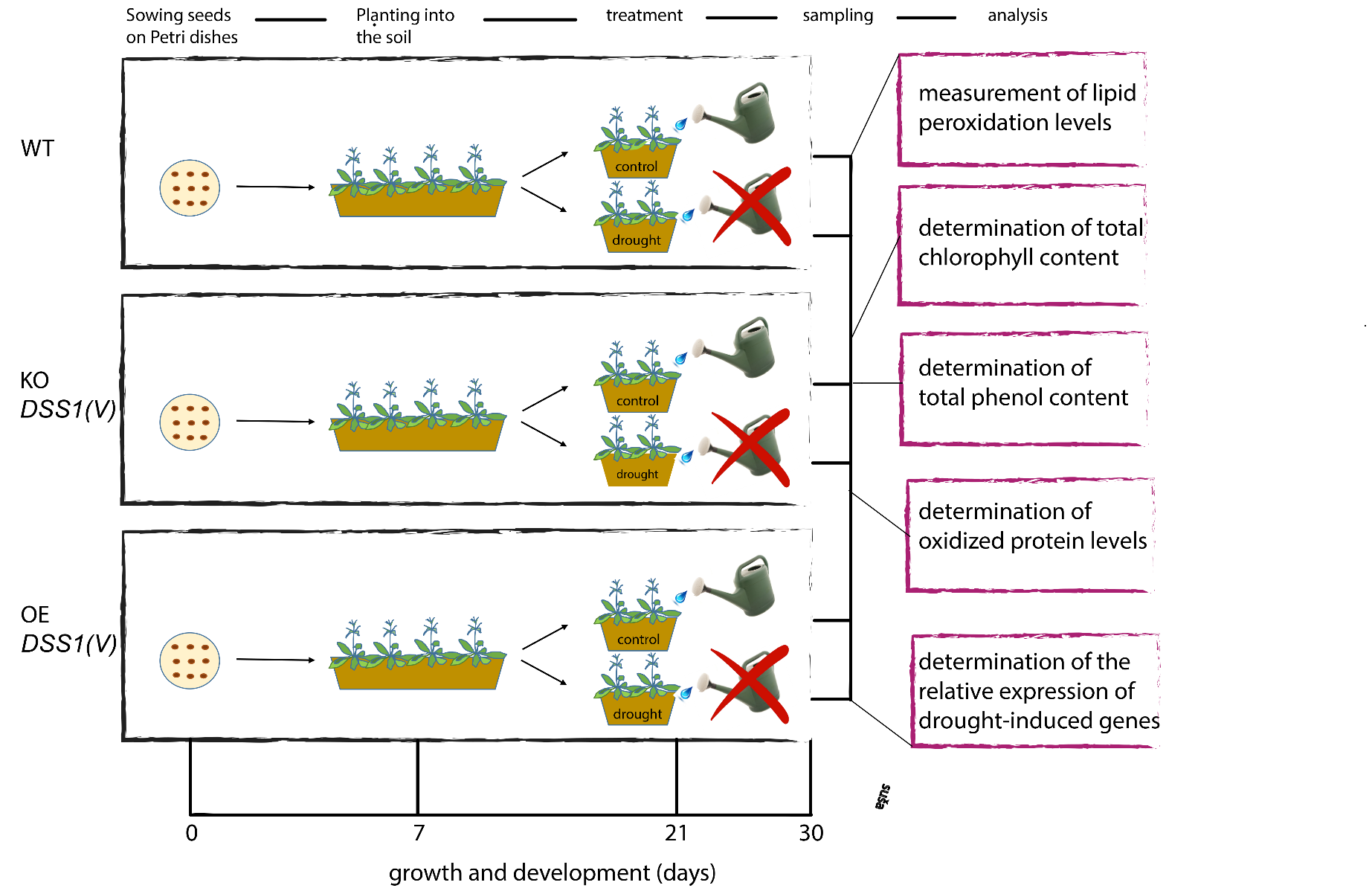


Figure S1: **Schematic representation of the experimental setup for drought treatment**.


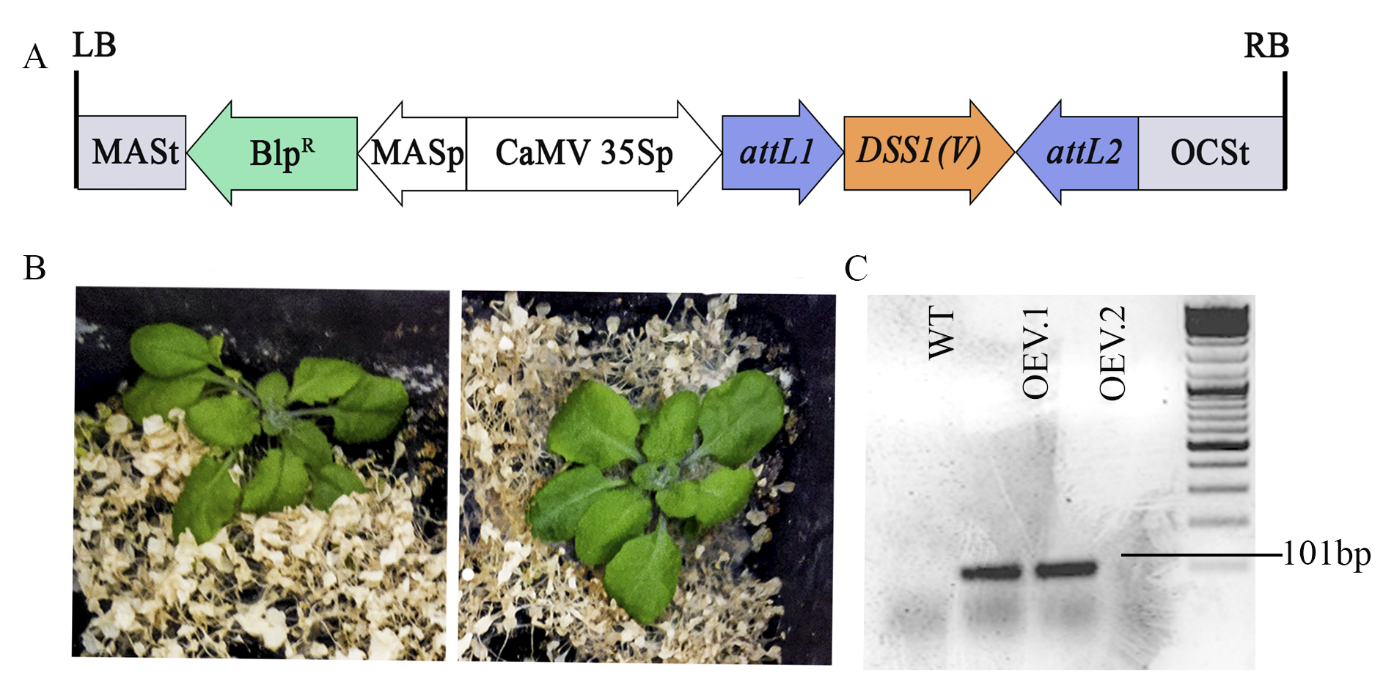


Figure S2. **Strategy for Generating Individual A. thaliana Lines with Enhanced Expression of DSS1(V). (A)** Schematic representation of the T-DNA insert in the binary vector pEarlyGate 100-DSS1(V): RB/LB – border sequences of the T-DNA insert; MASt – mannopine synthase terminator; BlpR – gene conferring resistance to Basta herbicide; MASp – mannopine synthase promoter; CaMV 35Sp – strong constitutive promoter from cauliflower mosaic virus 35S; attL1/attL2 – recombination sites for the Gateway BP reaction; DSS1(V) – cDNA sequence of the gene of interest; OCSt – octopine synthase terminator. **(B) T**wo transgenic plants OE DSS1(V).1, and OE DSS1(V).2, acquired following Basta treatment. **(C)** Detection of CaMV 35S promoter presence using PCR with primers 35Sf/35Sr on genomic DNA (gDNA) samples from WT plants and OE DSS1(V).1 and OE DSS1(V).2 (labeled OE(V).1 and OE(V).2 in the figure).


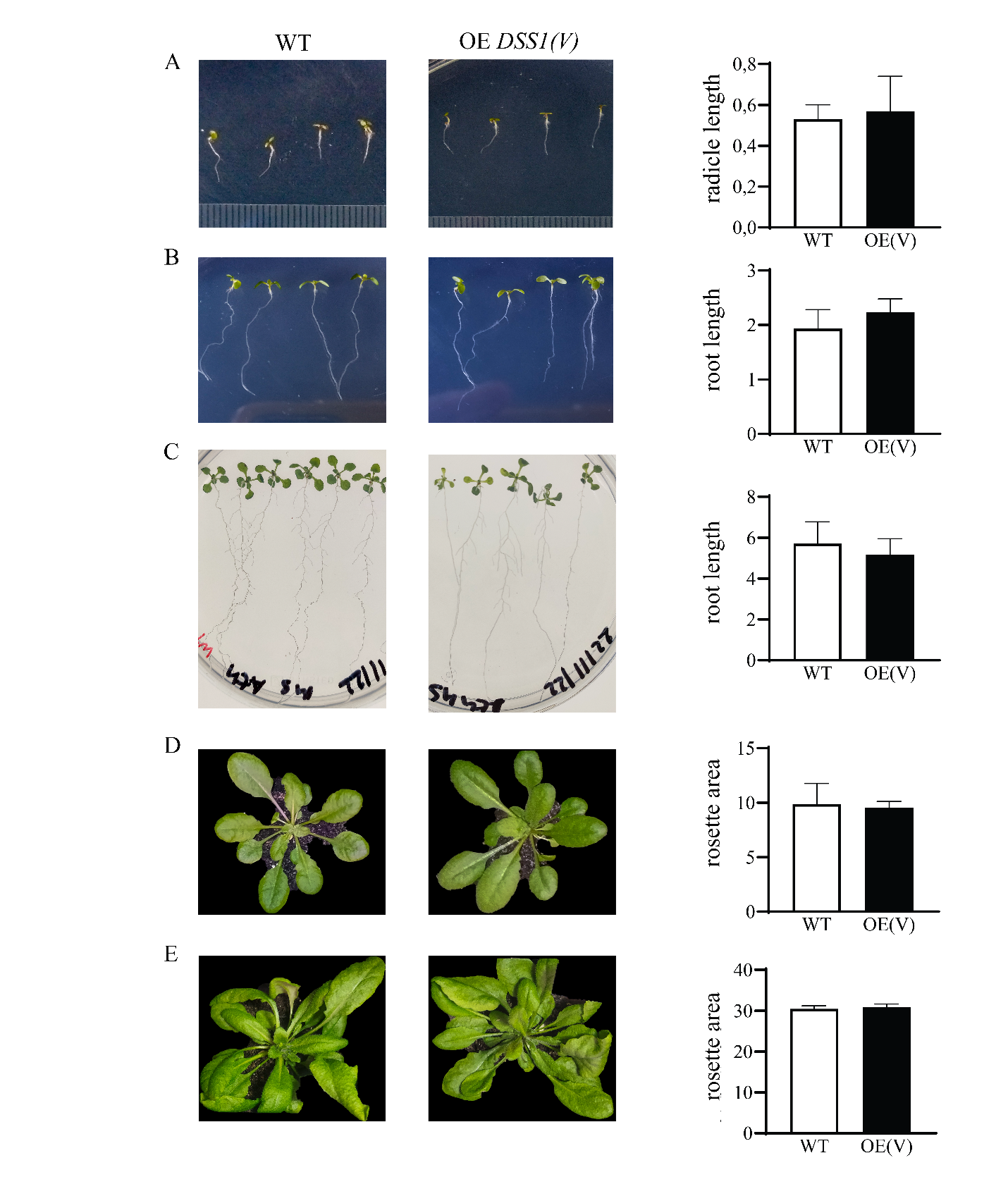


Figure S3. **Comparative Analysis of the Phenotype of WT and OE *DSS1(V)* *A. thaliana* Lines During Development.** After two weeks of germination and growth on MS medium in Petri dishes, seedlings were transfered into soil. (**A**) Photographs of three-day-old WT and OE *DSS1(V)* seedlings in Petri dishes; the histogram represents radicle length in cm. (**B**) Photographs of seven-day-old seedlings in Petri dishes; the histogram represents seedling length in cm. (**C**) Photographs of 14-day-old seedlings in Petri dishes; the histogram represents seedling length in cm. (**D**) Photographs of 24-day-old seedlings in soil; the histogram represents rosette area in cm². (**E**) Photographs of seven-week-old plants in soil; the histogram represents rosette area in cm². White bars correspond to measurements of various parameters in WT plants, while black bars represent OE *DSS1(V)* plants. Representative phenotypes of the plants are shown. Results are presented as mean ± SD, obtained from three independent biological replicates (n = 20 per experiment).
